# Focus on pattern recognition receptors to identify prognosis and immune microenvironment in colon cancer

**DOI:** 10.3389/fonc.2022.1010023

**Published:** 2022-09-23

**Authors:** Pengtao Ren, Yuan Zhang

**Affiliations:** ^1^ Department of Colorectal Anal Surgery, The Second Hospital of Hebei Medical University, Shijiazhuang, China; ^2^ Electrocardiogram Room, The Second Hospital of Hebei Medical University, Shijiazhuang, China

**Keywords:** pattern recognition receptors, colon adenocarcinoma, prognosis, tumor microenvionment, immunotherapy

## Abstract

In 2011, J. Hoffman, and B. Beutler won the Nobel Prize of medicine for the fact that they discovered the pattern recognition receptors (PRRs) and meanwhile described their effect on cell activation from the innate and adaptive immune systems. There are more and more evidences that have proved the obvious effect of PRRs on tumorigenesis progression. Nevertheless, the overall impact of PRR genes on prognosis, tumor microenvironmental characteristics and treatment response in patients with colon adenocarcinoma (COAD) remains unclear. In this research, we systematically assessed 20 PRR genes and comprehensively identified the prognostic value and enrichment degree of PRRs. The unsupervised clustering approach was employed for dividing COAD into 4 PRR subtypes, namely cluster A, cluster B, cluster C and cluster D, which were significantly different in terms of the clinical features, the immune infiltrations, and the functions. Among them, cluster B has better immune activities and functions. Cox and LASSO regression analysis was further applied to identify a prognostic five-PRR-based risk signature. Such signature can well predict patients’ overall survival (OS), together with a good robustness. Confounding parameters were controlled, with results indicating the ability of risk score to independently predict COAD patients’ OS. Besides, a nomogram with a strong reliability was created for enhancing the viability exhibited by the risk score in clinical practice. Also, patients who were classified based on the risk score owned distinguishable immune status and tumor mutation status, response to immunotherapy, as well as sensitivity to chemotherapy. A low risk score, featuring increased tumor stemness index (TSI), human leukocyte antigen (HLA), immune checkpoints, and immune activation, demonstrated a superior immunotherapeutic response. According to the study results, the prognostic PRR-based risk signature could serve as a robust biomarker for predicting the clinical outcomes as well as evaluating therapeutic response for COAD patients.

## Introduction

Colon cancer refers to the malignant lesions of colonic mucosa epithelium under the action of multiple carcinogenic factors such as environment or heredity, and is one of the common malignant tumors of digestive tract (1). Globally, about 8 million new cases occur each year, accounting for 10% to 15% of all malignancies (2). The colon adenocarcinoma (COAD) is the representative subtype regarding colon cancer, which takes up 98% of new colon cancer cases, with the 5-year survival rate of 40 – 60% (3). The early diagnosis rate of COAD was low, and most patients were in the middle and late stages when they were found (4). With the development of diagnostic techniques and optimization of treatment technologies, the mortality rate of COAD has decreased by 20% in the last 10 years (5). However, the exploration of new therapeutic targets and prognostic biomarkers for COAD at the molecular level still needs to be further carried out.

Innate immune pattern recognition receptors (PRRs) are crucial components of innate immunity, and constitute a bridge between the innate and acquired immunities (6). The immune system can remarkable affect the cancer formation, and it is essential to deeply understand the effect of specific PRRs in the cancer environment. In the past two decades, with the successive reports of various PRRs including TLR-like receptors, RLR-like receptors and NOD-like receptors, the research on innate immunity in cancer has set off a wave of climax (7, 8). PRRs can significantly regulate the tumor suppression and promote tumor cell responses in many cancer types. PRRs are capable of promoting the cancer shaping, to be specific, TLR9 activation by mitochondrial DNA in a hypoxic environment can induce the growth of hepatocellular carcinoma cells (9); TLR2 takes charge of triggering MyD88-IRAK1 signaling in the epithelial cells of breast cancer for inducing cell proliferation (10); activation of TLR2, TLR4, TLR7, TLR9 and NLRP3 can trigger multiple pro-tumor activities in pancreatic cancer (11). Besides, studies also proved the inhibiting effect of many PRRs on tumor progression, e.g., activating TLR8 in tumor cells is capable of preventing the generation of the immunosuppressive metabolite cAMP, thereby reversing immunosuppression in the tumor microenvironment (12); in colorectal cancer, TLR2, NOD1 and NLRP3 in the immune cells facilitate the antitumor activity *via* triggering the inflammation (13). However, there are no studies that confirm if the expression regarding PRR family-related genes are correlated with the COAD prognosis in clinical practice and if they can serve as the biomarkers of COAD.

Here, we provide a comprehensive overview of PRR-related genes in COAD and explore the potential of PRR signature for prognostic prediction and immunotherapeutic reflection.

## Materials and methods

### Dataset and preprocessing

The fragments per kilobase of transcript per million mapped reads (FPKM) format RNA sequencing (RNA-seq) data with complete follow-up information of 438 samples were downloaded from TCGA-COAD cohort. We excluded duplicate sequencing samples from the same patients and patients lacking complete follow-up information and with 0 survival days. We performed log2 [transcripts per million (TPM) + 1] transformation on the above raw data (14). Using the same exclusion criteria, 556 patients with COAD from the GEO database (GSE39582 cohort) were included as the validation cohort. Second, “sva” R package assisted in removing their batch effects (**Figure S1**).

### Exploring the function of PRR

Twenty PRR genes were included from previous literature. In the TCGA-COAD cohort, each tumor sample was scored for PRR status using the GSVA algorithm. In all PRR, the best cut-off value -0.6131245 was taken into account for grouping patients with high score and low score. In the two groups, we use “limma” package and | log2-fold change (FC) | ≥ 1 and p-value < 0.05 as the threshold for identifying DEGs.

### Unsupervised clustering analysis

Unsupervised consistent clustering analysis was performed based on the expression levels of DEGs. Principal component analysis (PCA) assisted in determining the independence between subtypes. The R package “ConsensuClusterPlus” helped to determine the cluster number and we performed 1000 iterations for ensuring their stability. Biological information was obtained from the KEGG database and GSVA assisted in evaluating the difference between subtypes in terms of the biological pathways.

### Risk score model construction and validation

LASSO regression analysis was used for removing redundant genes. Next, we used multivariate Cox regression analysis to further screen genes according to best AIC value. Finally, the gene expression values were first weighted by the LASSO-Cox coefficient which were then integrated for establishing the risk score formula. Cox regression analysis served for evaluating the independent prognostic value of risk score in training set and external validation set.

### Immune cell infiltration and tumour mutation burden estimation

For immune cell analysis, we used simultaneously different algorithms including TIMER, CIBERSORT, QUANTISEQ, MCP-counter, XCELL and EPIC for estimating the quantity and correlation of immune cells in various risk groups. Besides, the ssGSEA algorithm served for estimating immune cells as well as immune-related functions. MutSigCV algorithm assisted in selecting oncogenes of which the mutation frequency was higher relative to background, and maftools was used to display the mutations.

### Drug sensitivity analysis

The “prophetic” package in the R software assisted in the IC50 calculation. Chemotherapy drugs came from the Genome of Drug Sensitivity in Cancer (GDSC) database.

### qRT-PCR

TRIzol was employed to isolate the total RNA from COAD tumors as well as adjacent tissues, and SYBR Premix Ex Taq II (Takara, Shiga, Japan) served for cDNA amplification. Based on the three independent experiments, 2-^ΔΔCT^ values were applied for data analysis. The study has obtained the approval of The Ethics Committee of the Second Hospital of Hebei Medical University and obtained all patients’ written consent. For specific experimental protocols, please refer to previous studies in our hospital (15, 16).

### Statistical analysis

The R software (v.4.0.1) assisted in all the statistical analyses. Statistical methods for processing transcriptome data have been described in above section in detail. A p-value < 0.05 indicated statistical significance.

## Results

### Prognostic value of PRRs

**Figure 1:**
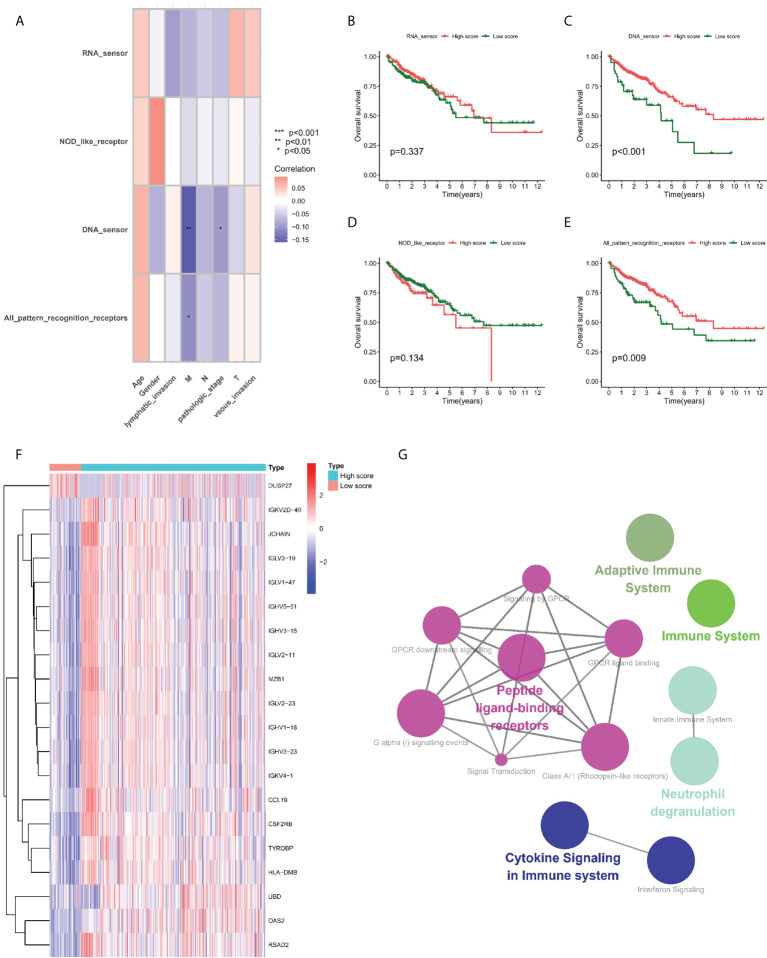
Prognostic value of PRRs. **(A)** Correlation between PRR enrichment scores and clinicopathological features. **(B–E)** Kaplan-Meier survival analysis between low-PRR enrichment scores and high-PRR enrichment scores. **(F)** Heatmap of DE-PRRs between low-PRR enrichment scores and high-PRR enrichment scores. **(G)** Enrichment analysis of DE-PRRs. *P < 0.05, **P < 0.01, ***P < 0.001.

GSVA algorithm assisted in calculating different PRR enrichment scores, and the correlation between the four groups of PRR enrichment scores and clinical pathological characteristics was further studied in the TCGA-COAD cohort. The results showed a negative correlation between partial PRR enrichment scores and M stage and pathological stage (**Figure 1A**). Subsequently, we performed survival analysis of four groups of PRR enrichment scores (**Figures 1B–E**), where the high and low scores in DNA sensor and all PRR were statistically different. We then grouped the TCGA-COAD cohort according to the cut off values in all PRR and explored the DEGs between the two groups. A majority of the DEGs were upregulated in the high score group, with 164 upregulated genes and 1 downregulated gene (**Figure 1F**). Finally, DEGs in clue GO underwent enrichment analysis, finding that entries such as peptide ligand-binding receptors, cytokine signaling in immune system, neutrophil deimmune system, immune system, and adaptive system were significantly enriched (**Figure 1G**).

### Clusters mediated by PRR related DEGs

**Figure 2:**
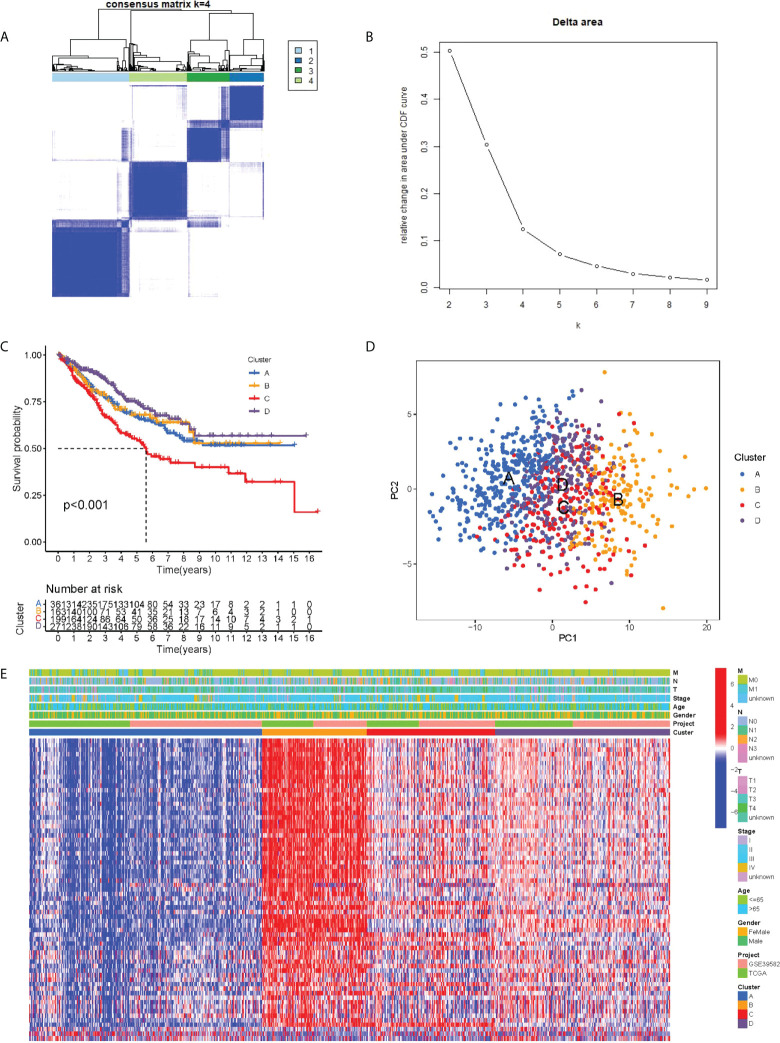
PRR subtypes. **(A)** Consensus clustering matrix when k = 4. **(B)** Relative change in area under the CDF curve for k = 2 through 9. **(C)** Kaplan-Meier curves of OS for four subtypes in COAD. **(D)** PCA analysis indicating an obvious difference in transcriptomes between the four subtypes. **(E)** Differences in clinicopathologic characteristics and expression levels of PRRs between the four distinct subtypes.

We integrated the clinical as well as transcriptome data regarding TCGA-COAD and GSE39582 cohorts into a meta cohort for further analysis. The expression levels of PRR-related DEGs were taken into account to classify patients into four subtypes under the assistance of the unsupervised consensus clustering analysis (**Figures 2A, B**), with cluster-A containing 361 patients, cluster-B containing 163 patients, cluster-C containing 199 patients, and cluster-D containing 271 patients. Based on the survival analysis, cluster C showed the worst prognosis among 4 clusters (**Figure 2C**). The PCA results demonstrated that at the transcriptome level, these four subtypes were relatively independent (**Figure 2D**). In addition, the heatmap showed the differential expression levels of PRR-related DEGs between clusters, demonstrating the distribution of relevant clinicopathological features, and it is worth noting that most genes in cluster B were significantly upregulated relative to other clusters (**Figure 2E**).

**Figure 3:**
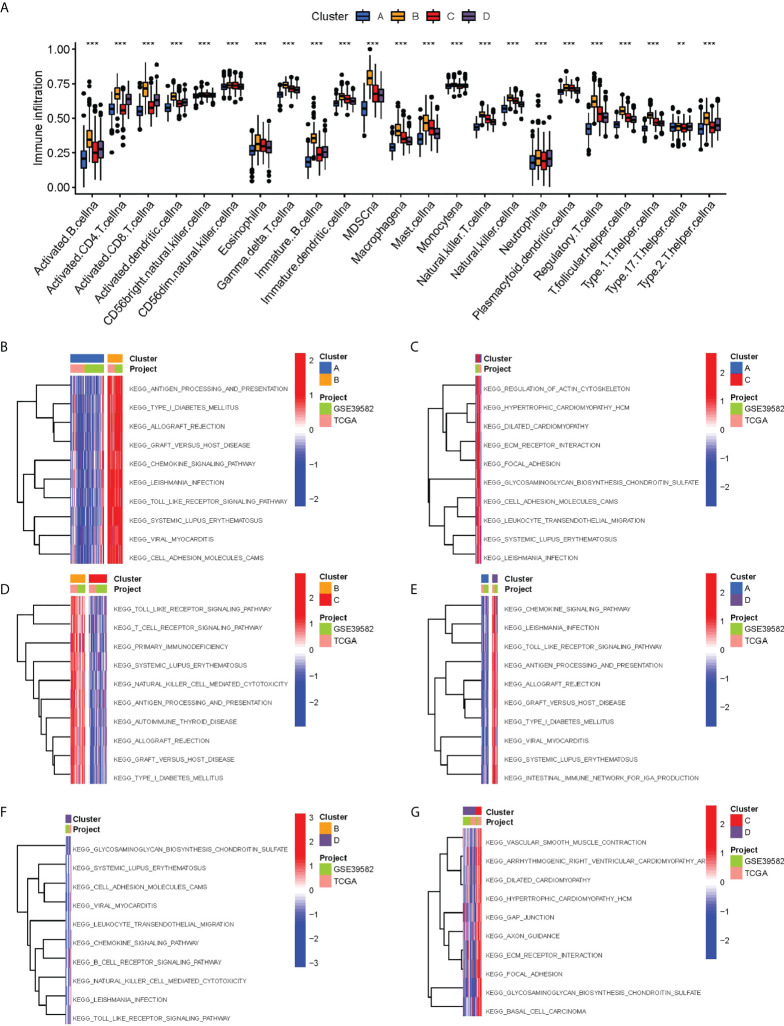
Biological characterization of molecular subtypes. **(A)** Differences in immune cell infiltration between different subtypes. **(B–G)** The GSVA pathway enrichment analysis between different subtypes.

The ssGSEA analysis showed most antigen-presenting cells and immune killer cells in cluster B were significantly upregulated (**Figure 3A**). To further explore the causes of immune microenvironment changes, the GSVA algorithm served for studying the biological process changes between the four clusters (**Figures 3B–G**). Most of the pathways in cluster B were significantly up-regulated compared with cluster A, such as ANTIGEN _ PROCESSING _ AND _ PRESENTATION, ALLOGRAFT _ REJECTION, HEMOKINE _ SIGNALING _ PATHWAY, TOLL _ LIKE _ RECEPTOR _ SIGNALING _ PATHWAY. In other two-by-two comparisons, cluster B also demonstrated a more abundant immune-related pathways active. Moreover, we used different algorithms (TIMER, CIBERSORT, MCP-counter, XCELL and EPIC) to estimate the abundance of immune cells based on the ‘IOBR package’. We found the result also showed the Cluster B maybe represent “hot tumor” (**Figure S2**).

### Construction and validation of risk model

**Figure 4:**
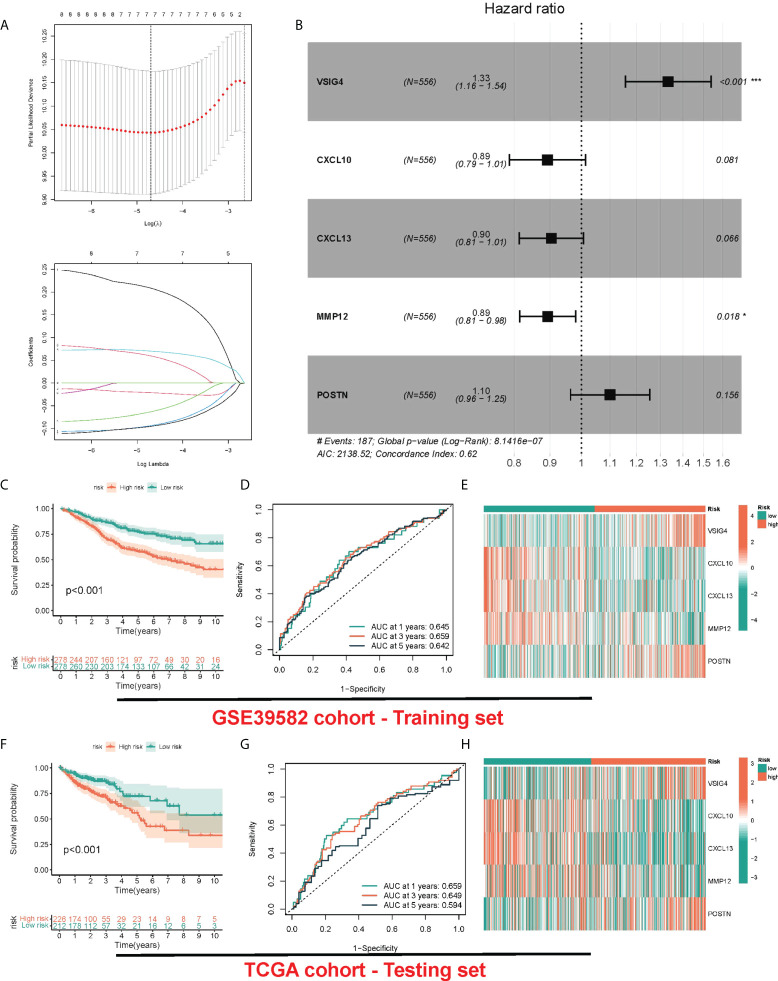
Construction and validation of risk model. **(A)** LASSO Cox regression analysis of PRRs. **(B)** Forest plot of the five target genes that compose the PRR signature. **(C, F)** KM survival analysis between low-risk and high-risk groups. **(D, G)** ROC curves analysis of PRR on OS at 1 year, 3 years, and 5 years. **(E, H)** Heatmap for the expression of five crucial genes in low-risk and high-risk groups.

Although the above molecular typing results are capable of predicting the difference of COAD patients in survival and function, the molecular typing mainly considers the patient group, so it is incapable of accurately predicting each patient’s risk status. Hence, based on the mRNA expression of PRR-related DEGs, we assessed riskscore for each individual patient for clinical application. In the GEO cohort, by the LASSO-Cox algorithm (**Figures 4A, B**), we finally obtained a risk score formula based on 5 genes: (0.2881 × expression level (EL)of VSIG4) + (-0.1126 × EL of CXCL10) + (-0.1000 × EL of CXCL13) + (-0.1121 × EL of MMP12) + (0.0952 × EL of POSTN). In addition, the same median risk score was taken into account for differentiating between patients in different risk groups in the TCGA-COAD cohort and the GSE39582 cohort. In the GSE39582 cohort, patients with high risk exhibited obviously lower OS relative to patients with low risk (**Figure 4C**). The area under the ROC curve (AUC) values were 0.645, 0.659, and 0.642 for 1-, 3-, and 5-year survival, respectively (**Figure 4D**). The heatmap showed up-regulated CXCL10, CXCL13 and MMP12 in the group with low risk, and up-regulated VSIG4 and POSTN in the group with high risk (**Figure 4E**). The risk score also showed similar prediction performance in the TGCA-COAD cohort, where the AUCs at 1, 3 and 5 years were 0.659, 0.649, and 0.594, respectively (**Figures 4F–H**). Moreover, we compared the risk signatures in other studies (17–19). The results were also exciting: the risk signature of our study showed better C-index value (**Figure S3**).

**Figure 5:**
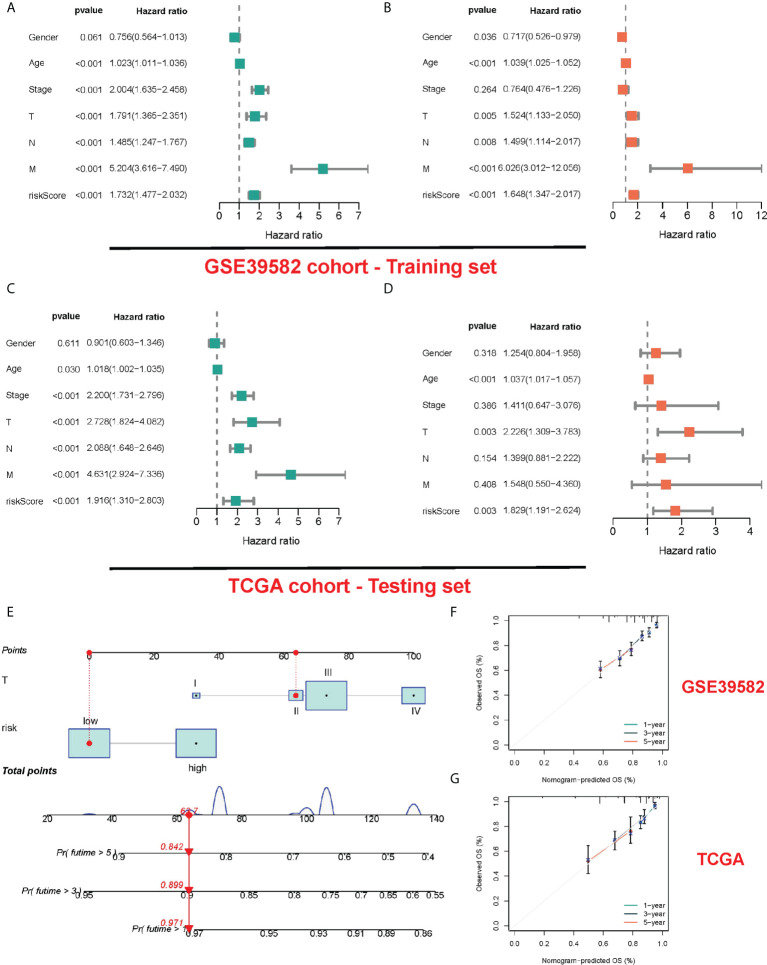
Construction and validation of a nomogram. **(A, B)** The results of the univariate and multivariate Cox regression analyses regarding significant survival-related clinical characteristic parameters in the GEO cohort. **(C, D)** The results of the univariate and multivariate Cox regression analyses regarding significant survival-related clinical characteristic parameters in the TCGA cohort. **(E)** The nomogram for predicting the survival probability of COAD patients. The calibration plots of the nomogram for predicting OS probability in GSE39582 cohort **(F)** and TCGA cohort **(G)**.

For determining whether risk score could independently predict COAD patients’ prognosis, Cox regression analysis was performed based on clinicopathological characteristics and risk score. As revealed by the univariate Cox regression analysis, in TCGA and GEO cohorts, the risk score is significantly correlated with OS (GEO cohort: HR = 1.732, 95% CI = 1.477-2.032; TCGA cohort: HR = 1.916, 95% CI = 1.310- 2.803) (**Figures 5A, C** **)**. After other confounding factors were adjusted, the risk score remained an independent predictor for COAD patients’ OS (GEO cohort: HR = 1.648, 95% CI = 1.347-2.017; TCGA cohort: HR = 1.829, 95% CI = 1.119-2.624) (**Figures 5B, D** **)**. The nomogram can directly serve for clinical work (**Figure 5E**). As found by the calibration curves, for both cohorts, the predicted curves were similar to the standard curves, which indicated the close relation between the predicted survival at 1, 3, and 5 years and the actual survival (**Figures 5F, G**).

### Immunity analysis

**Figure 6:**
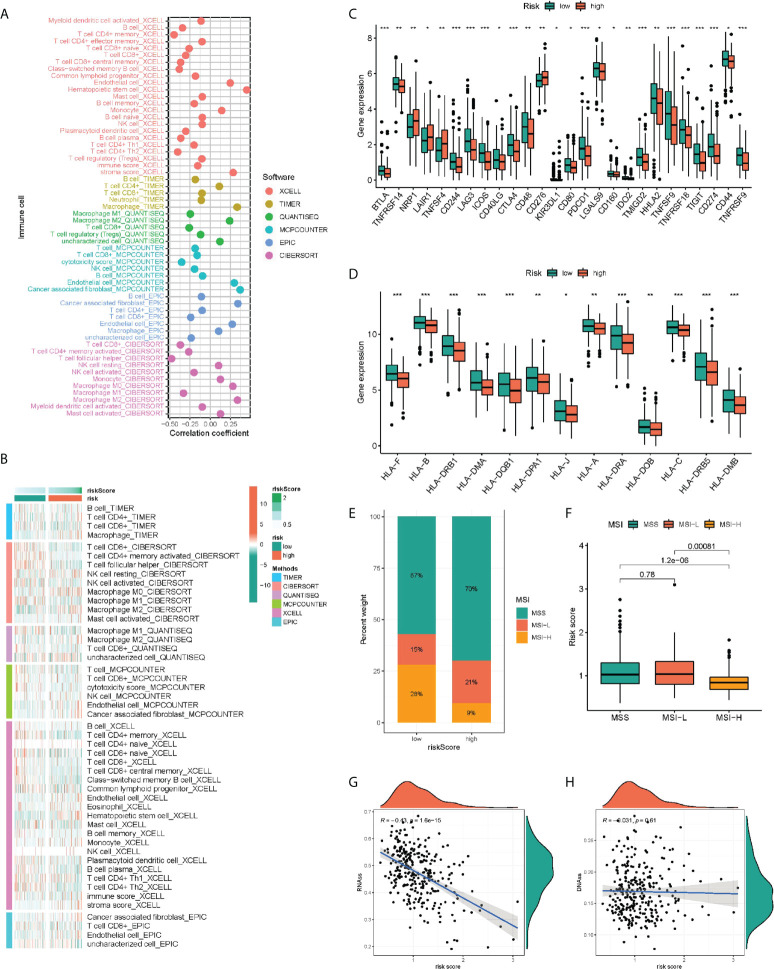
Immunity analysis of the PRR-related prognostic signature. The correlation of tumor-infiltrating cells with risk score using 6 algorithms. **(A)** Heatmap. **(B)** lollipop plot. **(C)** Expression of immune checkpoints in the high and low-risk groups. **(D)** Comparison of 13 HLA-related genes expression levels in two risk score subgroups. **(E–H)** The correlation of the risk score and MSI and TSI. *P < 0.05, **P < 0.01, ***P < 0.001.

For comprehensively exploring the association of risk subgroups with immune cell infiltration, six algorithms were adopted for plotting the correlation heatmap (**Figure 6A**) and lollipop plot (**Figure 6B**) regarding immune cell infiltration: TIMER, CIBERSORT, QUANTISEQ, MCP-counter, XCELL and EPIC. Interestingly, in various algorithms, most immune cells presented a negative relation to risk score. In addition, the immune function of low-risk patients was significantly activated, indicating that low-risk patients tended to be in hot tumor state, and they might respond better to immunotherapy. Given the importance of immunotherapy, we compared the two groups in terms of the expression levels regarding 26 candidate immune checkpoints, finding that most immune checkpoints presented a high expression in groups with low risk, such as PD-L1 and CTLA4 (**Figure 6C**). Similarly, human leukocyte antigen (HLA) was also significantly different, and the group with low risk presented higher expression (**Figure 6D**). We found that the risk score may also indicate the status of microsatellite instability (MSI), with a lower proportion of high microsatellite instability (MSI-H) in high-risk patients (**Figures 6E, F**). Finally, considering the effect of tumor stemness index (TSI) on tumor progression, we performed a correlation analysis between risk score and DNAss and RNAss (**Figures 6G, H**), in which RNAs decreased significantly with the increase of risk score.

### Mutation status in different risk subgroups

**Figure 7:**
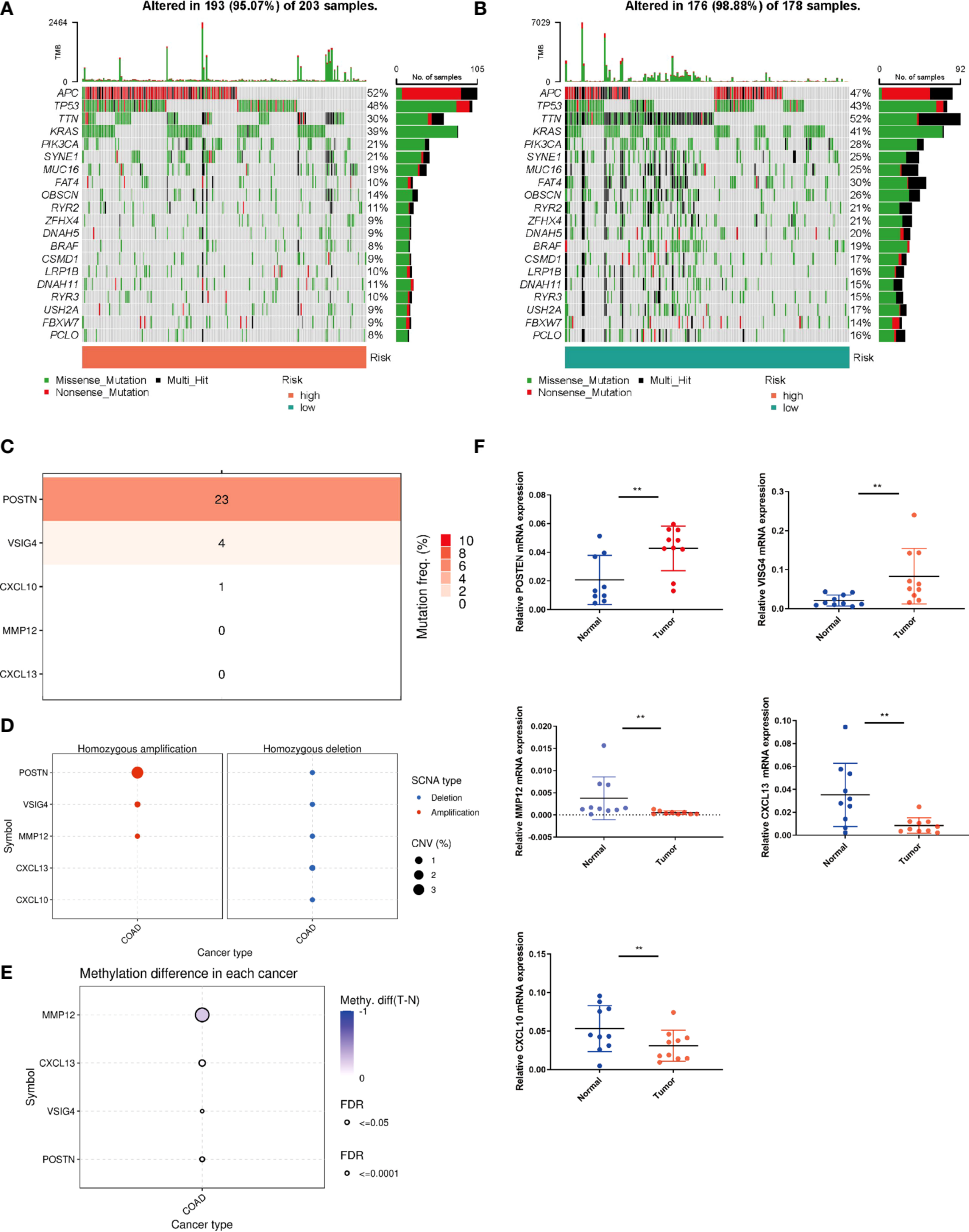
Mutation status in different risk subgroups. Waterfall maps of the somatic mutations in the high-risk group **(A)** and the low-risk group **(B)**. **(C)** Mutation rates of five genes (POSTN, VISG4, CXCL10, CXCL13, MMP12) in COAD patients. **(D)** Frequencies of CNV gain and loss among five PRRs. **(E)** Methylation analysis of four genes (POSTN, VISG4, CXCL13, MMP12) in COAD patients. **(F)** The expression levels of five PRRs in 10 paired COAD and matched adjacent normal tissues were examined by q-PCR. **P < 0.01.

We further analyzed the whole-exome sequencing data of patients with different risk groups, and found consistent high mutation genes in the two groups: APC, TP53, TTN, KRAS and PIK3CA (**Figures 7A, B**). In addition, we analyzed the five genes involved in the model construction in detail in the TCGA-COAD cohort, and found that the mutation frequency of POSTN was 23%, while that of MMP12 and CCL13 was 0 (**Figure 7C**). In CNV, POSTN also demonstrated the highest amplification (**Figure 7D**), and in the methylation level analysis, MMP12 was highly methylated (**Figure 7E**). Finally, we performed qPCR validation in clinical tissue samples and showed highly expressed POSTN, VISG4 in tumor samples, and highly expressed CXCL10, CXCL13, and MMP12 in normal samples (**Figure 7F**).

### Drug effectiveness analysis

**Figure 8:**
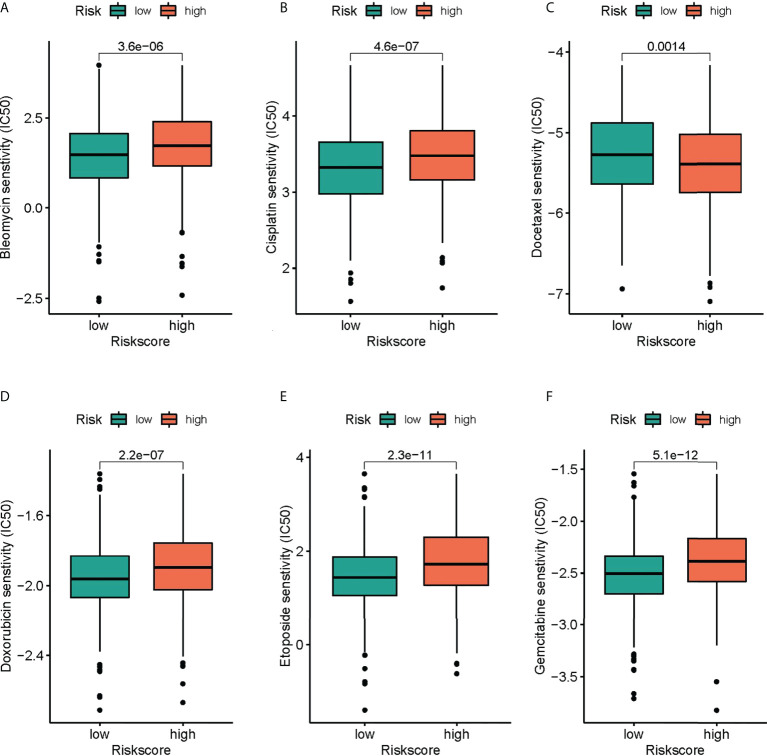
The differences in the chemotherapy response of common chemotherapy drugs between the high- and low-risk groups. **(A)** bleomycin, **(B)** cisplatin, **(C)** docetaxel, **(D)** doxorubicin, **(E)** etoposide, **(F)** gemcitabine.

GDSC served for comparing patients’ chemotherapy response to the common chemotherapy agents in the two groups (**Figures 8A–F**). The IC50 values of six chemotherapeutic drugs in patients with COAD were quantified. Most of the drugs were statistically different between different risk groups, and the group with low risk was more sensitive to the above chemotherapeutic drugs.

## Discussion

The immune system remarkably affects cancer shaping, from the early onset to the invasive metastasis and resistance to treatment (20). Since the significance of the immune system in the antitumour immunity has been recognized gradually, immune checkpoint inhibitors (ICIs) are applied in the immunotherapy for many cancers, despite the different efficacy (21). In comparison, innate immunity has not been applied in clinical practice. The dysregulation of innate immunity shows a relation to 1/3 of cancers and drives the the initiation and the maintenance regarding a chronic inflammatory state in the tumor microenvironment (TME), which is present throughout almost every stage of cancer development and cancer treatment resistance (22). In the past two decades, PRRs has gradually developed and crucially regulated the immune response to the microbial infection and the host tissue damage. In recently years, researchers have found the crucial effect exerted by PRRs on the modulation of many cellular responses regarding tumor inhibition and tumor promotion in the immune cells in the TME and directly in the cancer cells. The immune and non-immune functions of PRRs depend on the type of cancer (23, 24). Nevertheless, there are no studies that clearly explain how PRRs affect the clinical outcome, TME, and immunotherapy in COAD.

Twenty PRR genes reported in public were collected in the study. In the TCGA-COAD cohort, each tumor sample was scored for PRR status using the GSVA algorithm. In all PRR, the best cut-off value -0.6131245 was taking into account for dividing paints in group with high score and group with low score. In the two groups, “limma” package and | log2-fold change (FC) | ≥ 1 and p-value < 0.05 served as the threshold for identifying DEGs. It was found that most of the DEGs presented upregulation in the high score group, with 164 upregulated genes and 1 downregulated gene. Finally, DEGs in clue GO underwent enrichment analysis, finding that entries such as peptide ligand-binding receptors, cytokine signaling in immune system, neutrophil deimmune system, immune system, and adaptive system were significantly enriched. The unsupervised clustering approach was employed for dividing COAD into 4 PRR subtypes, namely cluster A, cluster B, cluster C and cluster D, which were significantly different in terms of the clinical features, the immune infiltrations, and the functions. Among them, cluster B has better immune activities and functions.

Although the above molecular typing results are capable of predicting the difference of COAD patients in survival and function, the molecular typing mainly considers the patient group, so it is incapable of accurately predicting each patient’s risk status. Hence, based on the mRNA expression of PRR-related DEGs, we assessed riskscore for each individual patient for clinical application. In the GEO cohort, by the LASSO-Cox algorithm, we finally obtained a risk score signature based on 5 genes. The signature classified COAD patients into group with low risk and group with high risk, and two independent validation cohorts verified its good performance and robust predicting efficiency regarding COAD survival. The signature was proved to be capable of well differentiating patients in different risk groups. Based on our study, risk score resulted from risk signature can independently predict OS. Besides, the PRR-based risk score was integrated with clinical factors, assisting in the construction of a nomogram, of which the efficacy was explained in calibration curves.

The immune system plays an important role in shaping all aspects of cancer, throughout the early initiation stage, tumor metastasis, and resistance to anti-cancer treatment. Humans have a deep understanding of the role of adaptive immunity in anti-tumor immunity and have developed immune checkpoint inhibitors (ICBs) for cancer immunotherapy. However, ICBs have different therapeutic effects in a variety of cancers (25). In contrast, innate immune function in cancer has not been fully utilized in clinic, although innate immune dysfunction is an important feature of all cancers. Recently, scientists have found that PRRs play a key role in regulating tumor cell response in many types of cancer. PRRs can play a role in immune cells and cancer cells in tumor microenvironment (26, 27). PRR provides a new perspective for clinical treatment of cancer. For a comprehensive exploration of the relation of risk subgroups to immune cell infiltration, six algorithms were employed for plotting the correlation heatmap and lollipop plot regarding immune cell infiltration: TIMER, CIBERSORT, QUANTISEQ, MCP-counter, XCELL, and EPIC. Interestingly, in various algorithms, most immune cells exhibited a negative relation to risk score. In addition, the immune function of low-risk patients was significantly activated, indicating that low-risk patients tended to be in hot tumor state, and they might respond better to immunotherapy. Given the importance of immunotherapy, we compared the two groups in terms of the expression level regarding 26 candidate immune checkpoints, finding that most immune checkpoints presented high expressions in group with low risk, such as PD-L1 and CTLA4. Similarly, HLA was also significantly different, which presented higher expression in the group with low risk. TSI and MSI, as the key biological markers for ICI response, can predict the immunotherapy response of various tumour types. More and more evidences found the higher sensitivity of high TSI/MSI patients to the immunotherapy (28, 29). In our study, MSI-H in group with low risk occupied a higher proportion. We analyzed the correlation of risk score with DNAss and RNAss, in which RNAs decreased significantly with the increase of risk score. Group with low risk reported obviously better clinical results relative to group with high risk, suggesting risk score could serve for independently predicting the responsiveness exhibited by immunotherapy.

This study has several limitations. Firstly, the findings were constructed and validated retrospectively in public databases. Therefore, it is necessary to conduct extensive prospective studies and supplementary *in vivo* and *in vitro* experimental studies to confirm our findings. Although there is significance in predicting the response to immunotherapy, this requires validation in another cohort of COAD patients undergoing immunotherapy.

To sum up, the study has confirmed the PRRs-based molecular subtypes in COAD, using PRRs for constructing a prognostic signature. In addition, patients with different risk score had different immune landscape, gene mutation status, expression of immune checkpoints, and drug sensitivity. Thus, PRR was a promising biomarker providing prognostic prediction and immune characterization, which may provide new strategies for personalized treatment in COAD patients.

## Data availability statement

The original contributions presented in the study are included in the article/**Supplementary Material**. Further inquiries can be directed to the corresponding author.

## Ethics statement

The studies involving human participants were reviewed and approved by The Ethics Committee of the Second Hospital of Hebei Medical University. The patients/participants provided their written informed consent to participate in this study.

## Author contributions

PR and YZ downloaded the dataset, analyzed the data, and wrote the manuscript. YZ reviewed the manuscript. All authors read and approved the final manuscript.

## Funding

This study was supported by Medical Science Research Plan Project of Hebei Province (No. 20200879).

## Conflict of interest

The authors declare that the research was conducted in the absence of any commercial or financial relationships that could be construed as a potential conflict of interest.

## Publisher’s note

All claims expressed in this article are solely those of the authors and do not necessarily represent those of their affiliated organizations, or those of the publisher, the editors and the reviewers. Any product that may be evaluated in this article, or claim that may be made by its manufacturer, is not guaranteed or endorsed by the publisher.

## Supplementary material

Supplementary Figure 1 The PCA plot of meta cohort before and after removing batch effects.

Supplementary Figure 2 Comparison of Immune cell content of different molecular subtypes in different algorithm.

Supplementary Figure 3 C-index in different risk signature.
