# Supplementary figures and images for: Focus on pattern recognition receptors to identify prognosis and immune microenvironment in colon cancer
Source: Front Oncol. 2022 Sep 23;12:1010023. doi: 10.3389/fonc.2022.1010023 (PMC9539811; doi:10.3389/fonc.2022.1010023)

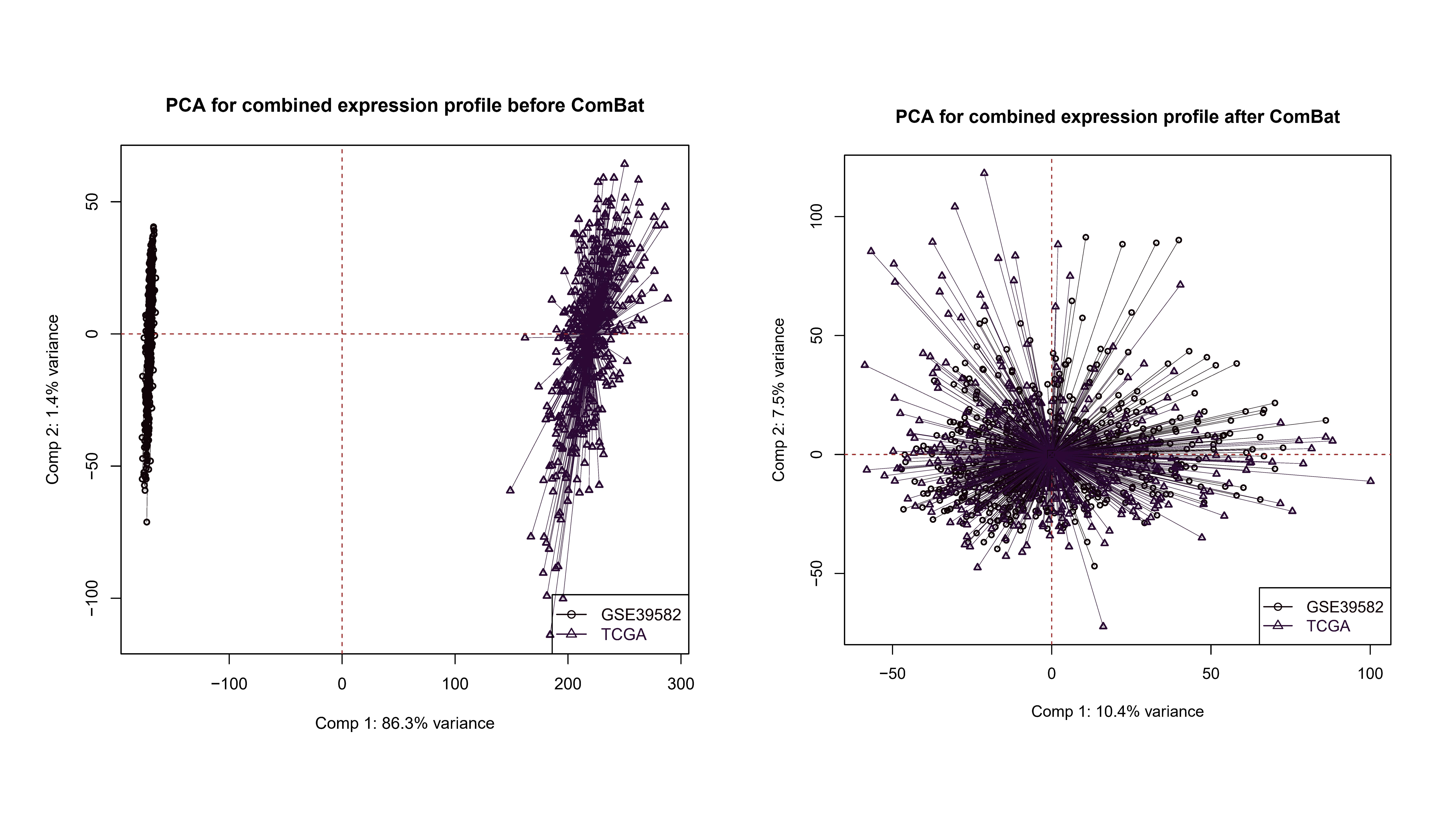

Supplement: Supplementary Figure 1 — The PCA plot of meta cohort before and after removing batch effects. [file Image_1.tif]

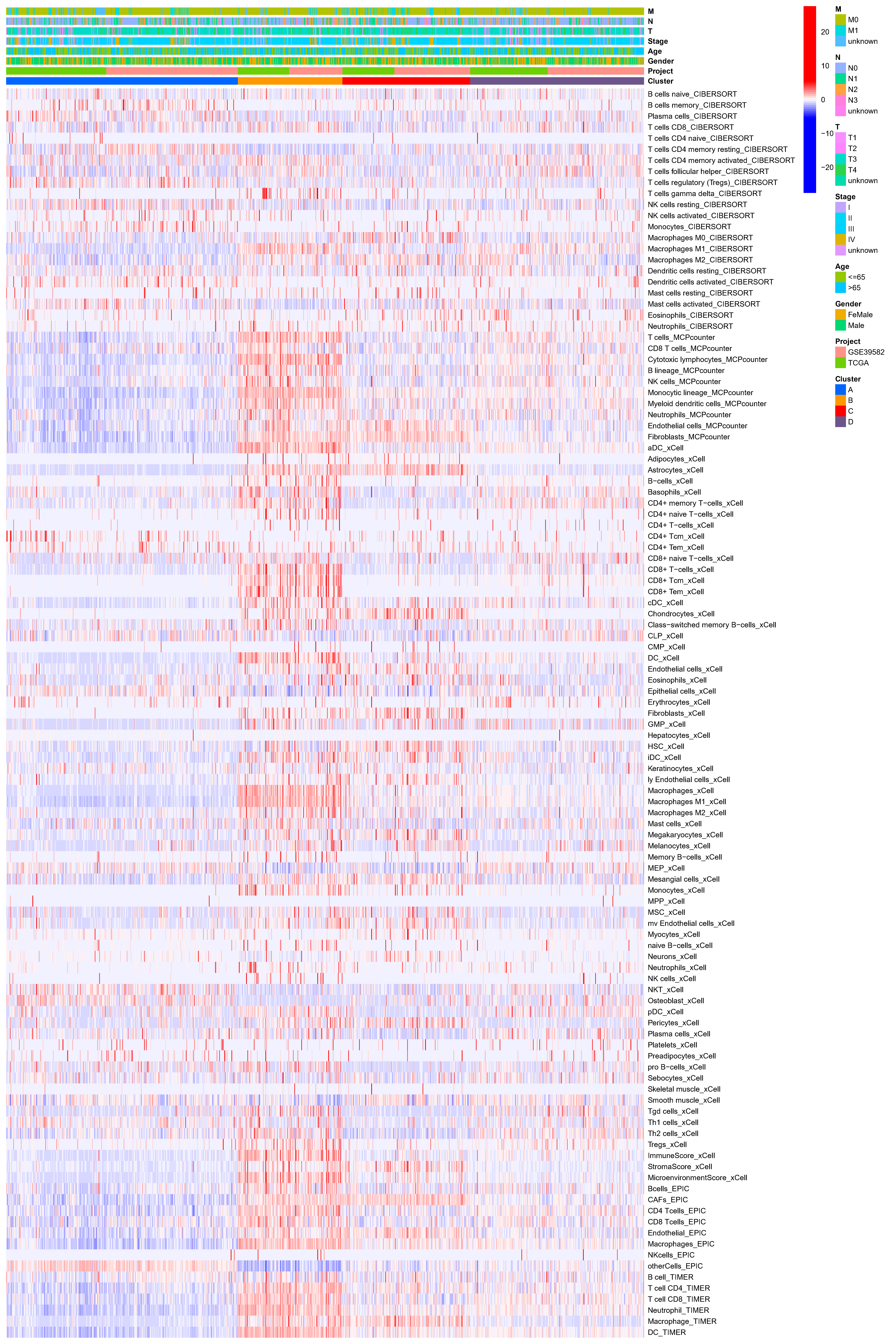

Supplement: Supplementary Figure 2 — Comparison of Immune cell content of different molecular subtypes in different algorithm. [file Image_2.tif]

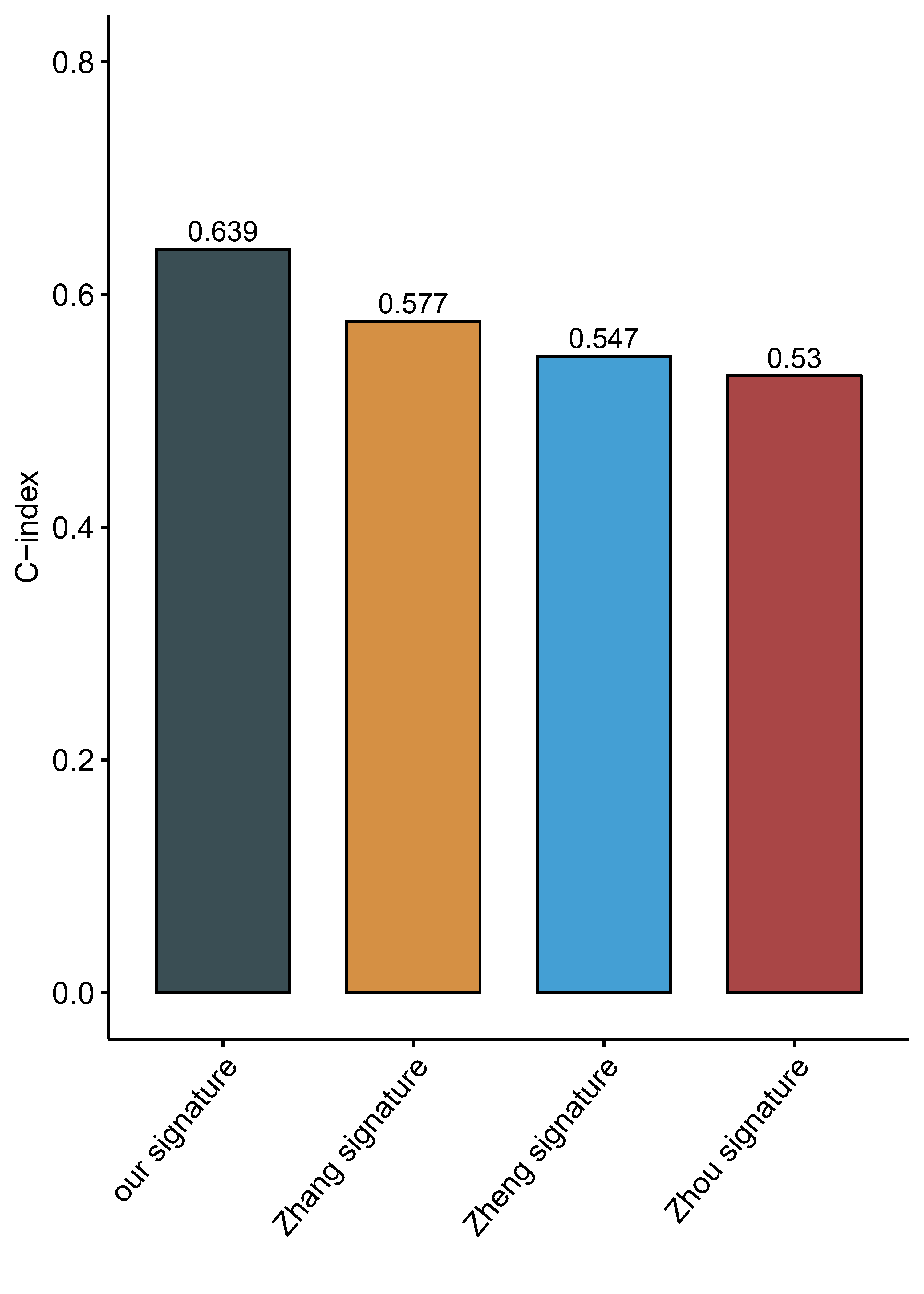

Supplement: Supplementary Figure 3 — C-index in different risk signature. [file Image_3.tif]
